# Evaluation of 3 and 2-point internal fixation in the management of zygomaticomaxillary complex fractures: Case report

**DOI:** 10.1016/j.amsu.2021.102539

**Published:** 2021-07-03

**Authors:** Dini Widiarni Widodo, Dwi Juliana Dewi, Respati Wulansari Ranakusuma, Yunia Irawati

**Affiliations:** a Department of Otorhinolaryngology, Head and Neck Surgery, Faculty of Medicine Universitas Indonesia, Cipto Mangunkusumo Hospital, Jakarta, Indonesia; b Clinical Epidemiology and Evidence-Based Medicine Unit, Faculty of Medicine Universitas Indonesia, Cipto Mangunkusumo Hospital, Jakarta, Indonesia; c Department of Ophthalmology, Faculty of Medicine Universitas Indonesia, Cipto Mangunkusumo Hospital, Jakarta, Indonesia

**Keywords:** Zygomatic fracture, Zygomaticomaxillary fracture, Internal fixation, Case report

## Abstract

**Introduction:**

The ZMC has a prominent shape compared to other parts in the midfacial region, thus small injuries will generate fractures in the ZMC. The management of ZMC fracture depends on the fracture deformity and the surgeon's considerations. Various studies have revealed the success of ZMC reconstruction with one fixation point to 4 fixation points fitting to the tetrapod shape.

**Case report:**

We report two cases of ZMC fractures which comparing the efficacy of 3- and 2-point internal fixations for improving clinical outcomes The first patient underwent ORIF which placed at 2 fixation points, the first point in the left ZF suture and the second point in the left ZMB. The second patient underwent ORIF reconstruction at 3 fixation points, the first point in the right inferior orbital rim, the second point in the right ZF suture, and the third point in the right ZMB.

**Discussion:**

The most common surgical approach for ZMC fractures is through a gingivobuccal groin incision. This approach is for body exposure of the ZMB, which is the main buttress. The 3-point internal fixation improved the postoperative clinical outcome of fracture fragment stability compared to two-point fixation, but the mean malar height projection, vertical dystopia, and enophthalmos were not different between the two fixation methods*.*

**Conclusion:**

Three-point internal fixation can improve the clinical outcome of fracture fragment stability compared to 2-point fixation; however, it has a mean operative duration 22.2 minutes longer than 2-point fixation, so its application must be considered during the COVID-19 pandemic.

## Highlights

• The incidence of ZMC fracture is reported to be 13%–40% of all facial fractures.
• Improper reduction of ZMC fracture can cause facial asymmetry and malposition.
• 3-point internal fixation improved the postoperative fracture fragment stability.
• 3-point internal fixation has a mean operative duration 22.2 minutes longer than the 2-point.

## 1 Introduction

The zygomaticomaxillary complex (ZMC) is the main buttress in the lateral midfacial region. The ZMC has a prominent shape compared to other parts in the midfacial region, thus small injuries will generate fractures in the ZMC. Fractures at the ZMC will also involve fractures of the bone forming the other midfacial region. The incidence of fracture in the ZMC is reported to be 13%–40% of all facial fractures. The most common causes of ZMC fractures are physical abuse and traffic accidents. It occurs more frequently in young adult males (ratio of males to females is 4:1) [1,2]

The zygomatic bone has a tetrapod-like shape, forming the midfacial regions as well as the horizontal buttress bones of the face. The ZMC forms the lateral part of the face together with the inferior wall of the orbit, which contributes to the malar projection and the width of the face. Contributing to its tetrapod-like shape are 4 points of articulation with other bones, namely, the zygomaticofrontal (ZF) suture line, zygomaticotemporal (ZT) suture line, zigomaticomaxillary buttress (ZMB), and zygomaticosphenoid (ZS) suture line. The suture line is an important benchmark in determining the fixation point to maintain the malar projection and reduce the fracture fragment. Fractures in the ZMC can occur in one or more tetrapod articulations. Improper reduction can cause significant malar flattening, facial asymmetry, changes in orbital volume, and malposition. Functional disorders that can occur include trismus and paraesthesia (due to pressure on facial nerves) [3].

The management of ZMC fracture depends on the fracture deformity and the surgeon's considerations. Various studies have revealed the success of ZMC reconstruction with one fixation point to 4 fixation points fitting to the tetrapod shape. In this case report, we describe our experience in the management of ZMC fracture using 2-point fixation and 3-point fixation. The importance of reporting this case lies in the rarity of clinical studies comparing the results of 2-point and 3-point fixation. This study was conducted to address this particular aspect in the management of ZMC fractures, so as to formulate an operative strategy that will achieve the surgical objective of stable fixation with better clinical results.

This work has been reported in line with the SCARE 2020 criteria [4].

### 1.1 Case report 1

The first patient was a 30-year-old female with a history of a traffic accident 6 months prior to surgery. The patient rode a motorcycle at moderate speed, and wore a half face helmet. The patient hit the car on the right side of the body and then fell to the left side with the left face facing the road. A few weeks after the accident, the patient has had a double vision, nasal congestion especially on the left side, impaired smell ability, and numbness on the cheeks.

**Fig. 1:**
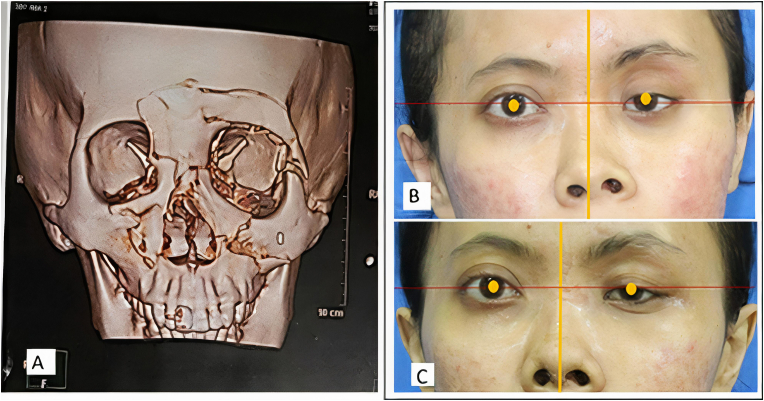
(A) Pre-surgery 3D facial CT-Scan; (B) Assessment of vertical dystopia by connecting the horizontal line between the two pupils and assessing the position of the pupils is not parallel in the horizontal plane. Preoperative facial radiograph showing the position of the pupil in a horizontal plane not parallel. (C) Postoperative facial photograph showing the position of the pupil in a more parallel horizontal plane.

Physical examination revealed the left nasal cavity was narrow and the septum was deviated to the left, contacting the inferior turbinate. The results of the nasal obstruction symptoms evaluation (NOSE) showed a value of 40 (moderate obstruction). External facial examination revealed a severe saddle nose with depressed nasal radix, tip deviation to the right, and a left malar depression (see Fig. 1).

Examination of the eye declared right eye visual acuity of 6/7.5, left eye visual acuity of 6/12, normal movement of both eyes in all directions, the distance between the intercanthal widened ± 5cm, left eye ptosis and left eye enophthalmos. Sensory abilities of the left V1, V2, and V3 nerves were decreased.

**Fig. 2:**
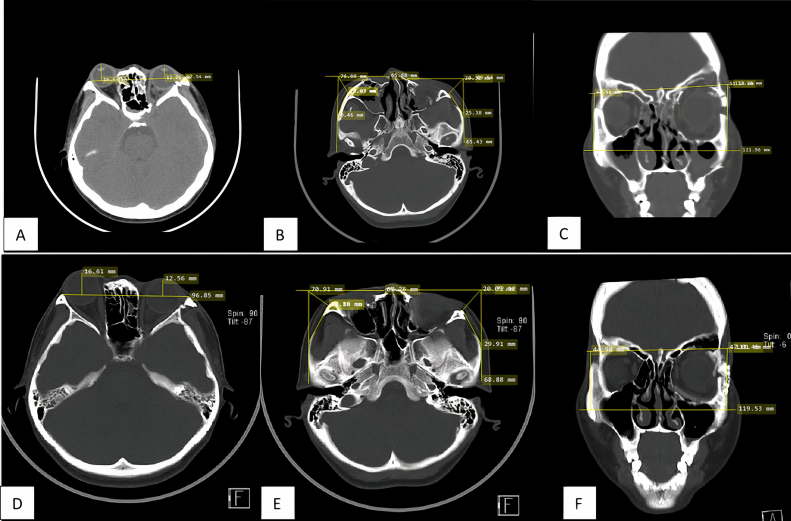
(A) Enophthalmos examination on preoperative CT scan using the Hilal and Trokel method shows enophthalmos in the left eye (with a difference of >2 mm between the two eyes). (B) Malar projection deficit examination. Assessed by axial sections, the width of the zygoma anteriorly and posteriorly was assessed by measuring the distance at that point. There is a deficit between the fracture side and the normal of 1.65 mm. (C) Malar height deficit examination. Coronal sections were assessed by connecting the horizontal line of the upper border of the orbit and the horizontal lines of the right and left zygoma arches. The difference is 7.03 mm compared to the normal side. (D) Enophthalmos examination on postoperative CT scan shows enophthalmos in the left eye (with a difference of >2 mm between the two eyes). (B) Malar projection deficit examination shows a decreasing deficit between the fracture side and the normal side of 1.27 mm. (C) Malar height deficit examination shows a decreasing deficit between the fracture side and the normal side of 2.03 mm.

A Facial Computed Tomography Scan (CT-Scan) revealed multifocal comminuted fractures of the bones of the maxillary sinus, ethmoid anterior sinus, and bilateral frontal sinuses and lateral orbital rims, with an impression component in part of the fracture fragment. Measurement of enophthalmos based on facial CT scan revealed left enophthalmos with a 3.82 mm difference from the right orbital. The measurement of the malar projection deficit showed a difference in the malar projection of 1.65 mm. The measurement of the malar height deficit shows a difference of 7.03 mm (see Fig. 2).

The patient was diagnosed with left ZMC fracture, frontonasal tripod fracture and orbital floor fracture. The patient underwent open reduction internal fixation (ORIF) reconstruction by the Facial Plastic Reconstruction Division, Otorhinolaryngology Head and Neck Surgery Department, Faculty of Medicine, Universitas Indonesia in a joint surgery with the Ophthalmology Reconstruction Division. The ORIF was placed at 2 points of fixation: the first point in the left ZF suture and the second point in the left ZMB. The patient was laid in the supine position, sedated with general anesthesia and intubated using endotracheal intubation. A subciliary incision was made on the left side. The inferior orbital rim was evaluated for fracture line with acceptable alignment. On the evaluation of the left orbital lateral wall, the fracture line was seen, followed by placement of plate and screw on the ZF suture. During the left sublabial incision, maxillary fracture was evaluated, and the plate and screw was placed on the left ZMB.

The patient then underwent post-operative treatment for 3 days, followed by outpatient post-operative wound care. At 3 months postoperative examination, there was improvement in vertical dystopia and nasal obstruction symptoms A facial CT scan after surgery revealed a decrease in malar projection deficits (the measurement shows a difference of 1.27 mm) and malar height deficits (the measurement shows a difference of 2.03 mm). Sign of enophthalmos was still visible from facial 3D CT-Scan. There was no post-operative complication. The patient was satisfied with the surgical outcome (see Fig. 2).

### 1.2 Case report 2

The second patient was an 18-year-old female patient with a history of a traffic accident 6 months prior to admission. The patient rode a motorbike at moderate speed, using a half face helmet. The direction of the trauma was from the left, causing the right side of the face to hit the ground. The patient experienced a closed right eyelid and numbness on the right cheek. Patient underwent wound reconstruction surgery of the right eyebrow and nose at Fatmawati Central General Hospital.

Physical examination revealed right and left nasal cavity within normal limits and no sign of nasal septal deviation were found. The results of the NOSE score showed a value of 10 (mild obstruction). On external facial examination, a right malar depression was found.

Examination of the eye revealed right eye visual acuity of 6/7.5, left eye visual acuity of 6/6, limited lateral movement of the right eye, normal left eye movement in all directions, diplopia, normal intercanthal distance, with right eye ptosis and enophthalmos.

**Fig. 3:**
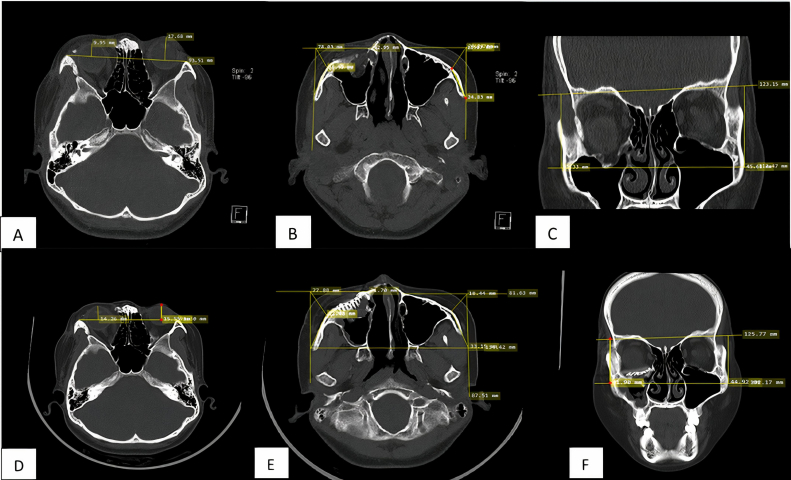
(A) Enophthalmos examination on preoperative CT scan shows enophthalmos in the left eye (with a difference of >2 mm between the two eyes). (B) Malar projection deficit examination. There is a deficit between the fracture side and the normal of 2.86 mm. (C) Malar height deficit examination. The difference is 4.35 mm compared to the normal side. (D) Enophthalmos examination on postoperative CT-scan shows improvement of enophthalmos in the right eye (with a difference of< 2mm between the two eyes). (E) Malar projection deficit examination shows decreasing deficit between the fracture side and the normal of 1,13mm. (F) Malar height deficit examination shows decreasing deficit between the fracture side and the normal of 3.02mm.

The results of facial CT-Scan imaging showed comminuted fracture of the right inferior orbital rim, right zygoma, and lateral wall of the right ethmoid sinus, accompanied by multiple bone fragments in the right intracavum orbital. This caused a prolapse of the periorbital tissue to the right maxillary sinus and was accompanied by displacement of the right orbital posteriorly. Examination of vertical dystopia was difficult to perform due to ptosis in the right eye. Measurement of enophthalmos based on facial CT scan revealed right enophthalmos with an 8.27 mm difference from the left orbital. The measurement of the malar projection deficit showed a difference in the malar projection of 2.86 mm. The measurement of the malar height deficit shows a difference of 4.35 mm (see Fig. 3).

The patient was diagnosed with right inferior orbital rim fracture, right os zygoma fracture, and fracture of the right ethmoid wall. The patient underwent ORIF reconstruction at 3 points of fixation. The first point in the right inferior orbital rim, the second point right ZF suture, and the third point in the right ZMB. The patient had a 3 mm subciliary incision under the right inferior eyelid, blunt dissection was performed until the periosteum was found, the trapped periorbital tissue was freed, then a silicon block and costal cartilage graft were placed in the defect area (right inferior orbit) followed by placement of mesh plate. A plate and screw were placed in the right inferior orbital rim. Evaluation towards the right superior zygomatic arch revealed an inline fracture line. A right sublabial incision was made, undermining superiorly until the periosteum and fracture line were identified. The plate and screw were placed on the ZMB. An incision was made in the right zygomaticofrontal area, an evaluation of the fracture line was performed, and a plate and screw were placed on the ZF suture.

A facial CT scan 3 months after surgery revealed improvement in enophthalmos (difference <2 mm between both eyes), a decrease in malar projection deficit (the measurement shows a difference of 1.13 mm), and decreased malar height deficit (the measurement shows a difference of 3.02 mm). In this patient, vertical dystopia could not be assessed due to uncorrected ptosis (see Fig. 3).

## 2 Discussion

The most common surgical approach for ZMC fractures is through a gingivobuccal groin incision. This approach is for body exposure of the ZMB, which is the main buttress. In certain cases, the use of a single 1.6 mm l-plate on the lateral part of the ZMC is sufficient to fix this section. In the case of a ZMC fracture accompanied by a zygoma arc fracture, the approach through a gingivobuccal groove incision can provide good exposure during reduction and stabilization of the region. In cases with orbital rim fractures, the approach is performed through a transconjunctival incision of the inferior palpebra. However, this approach can cause scarring of the palpebrae and malposition of the inferior palpebra. In general, the gingivobuccal incision is performed before the subciliar incision. In the case of the subciliar incision approach performed before the gingivobuccal incision, the zygoma-maxilla reconstruction process becomes more difficult as the fracture fragments are not in the correct anatomical position [3].

In comparing the type of fixation to the clinical outcome of patients with ZMC fracture, 3-point fixation gave better results in the assessment of vertical dystopia, enophthalmos, malar projection, malar height, and maintaining postoperative ZMC stability. In concordance with the results of the study presented by Parashar et al. [5] and Rana et al. [6], in a randomized controlled study, 3-point fixation achieved better postoperative fracture fragment stability than 2-point fixation. A single plate will theoretically stabilize the segment for translation and rotation in the 2 axis perpendicular to the plane of the plate. In some cases, this remaining motion can be neutralized by bony buttressing at the fracture interface. Otherwise, a second, appropriately positioned plate should be used to compensate for the remaining motion. A still more stable confirmation can be achieved by creating 3 fixation points that are not collinear. Given the constant biomechanical strain on the zygoma and the possibility of hardware failure, this 3-point could provide a safe construct that results in a significantly more stable fixation [7]. The 3-point fixations performed in the 3 studies were fixated on the ZF suture, inferior orbital rhyme, and on the ZM buttress. This is consistent with the research proposed by Farber et al. [2], in their study that the type of fixation selected in the treatment must be in accordance with the type of ZMC fracture and the involvement of 5 other points, namely, the ZF suture, inferior orbital rhyme, ZS suture, ZM buttress, and zygoma arc. Based on these anatomical conditions, the more fixation points applied, the better postoperative stability.

The biomechanical process of the maxillary bone and zygoma is an important basis in choosing the fixation technique that will affect postoperative stability. Latif et al. [8] reported in their study that at fixation of 3 points, the postoperative outcome (malar height prominence, vertical dystopia, and fracture fragment stability) had relatively the same mean values at the 1 week, 3 weeks, and 6 weeks postoperative follow-up, compared with 2-point fixation, which has a mean with a different relative range. In his research, 3-point fixation had an increasing mean malar height prominence with decreasing vertical dystopia and high stability. Bergeron et al. [9] reported that postoperative management of patients with ZMC fractures can extend up to 6 weeks postoperatively depending to the wound healing process. Therefore, follow-up up to 6 weeks postoperatively is particularly important to achieve functional stability and good aesthetics.

Another factor that affects postoperative stability is the type of plate used. Farber et al. [2] explained in their study that titanium plates have better stability in maintaining ZMC biomechanics. Karimi et al. [10] also argued similarly in their study; 3-point fixation in the management of ZMC fracture is the best strategy. It has been reported that using 2-point fixation increases the risk of postoperative complications such as decreased malar height prominence and vertical dystopia compared to 3-point fixation.

Despite the advantages, there were apparent disadvantages of 3-point fixation, such as extensive periosteal stripping, extreme retraction of bone edges and requirement of expert assistance for application of miniplate across the zygomatico-maxillary buttress, as well as longer operative time (approximately 22.2 minutes), presence of more hardware, and increased cost of surgery [5,6,11].

## 3 Conclusion

Three-point internal fixation can improve the postoperative clinical outcome of fracture fragment stability compared to 2-point fixation; however, the mean malar height projection, vertical dystopia, and enophthalmos are not different compared to 2-point internal fixation. Three-point internal fixation has a mean operative duration 22.2 minutes longer than 2-point fixation, so its application must be considered during the COVID-19 pandemic.

## Provenance and peer review

Not commissioned, externally peer reviewed.

## Sources of funding

The authors declared that this study has received no financial support.

## Ethical approval

This is a case report and there is no need for ethical committee approval. Nevertheless, informed consent is taken from patient.

## Consent

Written informed consent was obtained from the patient for publication of this case report and accompanying images. A copy of the written consent is available for review by the Editor-in-Chief of this journal on request.

## Author contribution

Dini Widiarni Widodo: study concept, data collection, data analysis, writing paper, final approval. Dwi Juliana Dewi: study concept, data collection, data analysis, writing paper, final approval. Respati Wulansari Ranakusuma: final approval Yunia Irawatifinal approval.

## Research registration

N/A.

## Guarantor

The Guarantor of this study is Dini Widiarni Widodo.

## Declaration of competing interest

Authors of this article have no conflict or competing interests. All of the authors approved the final version of the manuscript.
